# Fast and slow myofiber nuclei, satellite cells, and size distribution with lifelong endurance exercise in men and women

**DOI:** 10.14814/phy2.16052

**Published:** 2024-07-10

**Authors:** Cristhian F. Montenegro, Chad Skiles, Dillon J. Kuszmaul, Aaron Gouw, Kiril Minchev, Toby L. Chambers, Ulrika Raue, Todd A. Trappe, Scott Trappe

**Affiliations:** ^1^ Human Performance Laboratory Ball State University Muncie Indiana USA

**Keywords:** aging, cross‐sectional area, fiber type specific, masters athletes, myonuclei, satellite cells

## Abstract

We previously observed lifelong endurance exercise (LLE) influenced quadriceps whole‐muscle and myofiber size in a fiber‐type and sex‐specific manner. The current follow‐up exploratory investigation examined myofiber size regulators and myofiber size distribution in vastus lateralis biopsies from these same LLE men (*n* = 21, 74 ± 1 years) and women (*n* = 7, 72 ± 2 years) as well as old, healthy nonexercisers (OH; men: *n* = 10, 75 ± 1 years; women: *n* = 10, 75 ± 1 years) and young exercisers (YE; men: *n* = 10, 25 ± 1 years; women: *n* = 10, 25 ± 1 years). LLE exercised ~5 days/week, ~7 h/week for the previous 52 ± 1 years. Slow (myosin heavy chain (MHC) I) and fast (MHC IIa) myofiber nuclei/fiber, myonuclear domain, satellite cells/fiber, and satellite cell density were not influenced (*p* > 0.05) by LLE in men and women. The aging groups had ~50%–60% higher proportion of large (>7000 μm^2^) and small (<3000 μm^2^) myofibers (OH; men: 44%, women: 48%, LLE; men: 42%, women: 42%, YE; men: 27%, women: 29%). LLE men had triple the proportion of large slow fibers (LLE: 21%, YE: 7%, OH: 7%), while LLE women had more small slow fibers (LLE: 15%, YE: 8%, OH: 9%). LLE reduced by ~50% the proportion of small fast (MHC II containing) fibers in the aging men (OH: 14%, LLE: 7%) and women (OH: 35%, LLE: 18%). These data, coupled with previous findings, suggest that myonuclei and satellite cell content are uninfluenced by lifelong endurance exercise in men ~60–90 years, and this now also extends to septuagenarian lifelong endurance exercise women. Additionally, lifelong endurance exercise appears to influence the relative abundance of small and large myofibers (fast and slow) differently between men and women.

## 1 INTRODUCTION

The exercise boom of the 1960s and 1970s led to a generation of exercisers who are now in their 8th decade of life. We had the unique opportunity to study several men and women from this era that are now >70 years old and have had consistent endurance exercise habits in excess of 50 years. Several studies have been published on these cohorts highlighting the influence of lifelong endurance exercise (LLE) on cardiovascular health (Gries et al., 2018), whole muscle mass, function, and adiposity (Chambers et al., 2020), slow and fast myofiber function (Gries et al., 2019; Grosicki et al., 2021), skeletal muscle‐related inflammation (Lavin et al., 2020a, 2020b) and immunity (Perkins et al., 2020, 2024), and slow and fast myofiber transcriptome dynamics (Raue et al., 2023). We observed a partial preservation of quadriceps muscle mass in the LLE men, which was not observed in the LLE women (Chambers et al., 2020). At the single muscle fiber level, LLE men had substantially larger and more powerful slow muscle fibers with limited benefits for fast muscle fibers. In contrast, LLE women showed some benefit for contractile performance, but no benefit for slow or fast muscle fiber size.

The nuclei found on multinucleated skeletal muscle cells, and the pluripotent stem cells (i.e., satellite cells) that can replenish the myonuclear pool, have been investigated over the last six decades as regulators of skeletal muscle growth and atrophy (Dumont et al., 2015; Mauro, 1961; Murach et al., 2021; Snijders et al., 2015). A more recent focus has been on understanding skeletal muscle adaptations to aging and to exercise training in humans (Franco et al., 2019; Larsson et al., 2019; Thornell et al., 2003). In general, the human studies have focused on myonuclear number, myonuclear domain (i.e., the area “governed” by a myonucleus), and satellite cell number to gain insight into age and exercise training related muscle mass regulation.

The combination of endurance exercise training throughout the aging process (i.e., lifelong exercise) and the related influence on the myonuclear and satellite cell profile in human skeletal muscle has received little attention. The only two investigations in this area examined sexagenarian (Mackey et al., 2014) and octogenarian (Skoglund et al., 2023) men, both showing no apparent influence on myonuclear or satellite cell number. However, the sexagenarian lifelong runners exercised about half of the weekly exercise time of the septuagenarian men in our current study and the investigation of the octogenarian lifelong cross‐country skiers did not have a young comparator group. In addition, no women were studied in either investigation. Nonetheless, these two studies provide foundational insight into lifelong endurance exercise and the skeletal muscle myonuclei and satellite cell pools in humans. Yet, significant gaps in our understanding in this area still exist.

Considering our whole muscle and fiber type specific size findings in the LLE cohorts (Chambers et al., 2020;Gries et al., 2019; Grosicki et al., 2021), we conducted a follow‐up exploratory examination of slow and fast muscle fiber myonuclei and satellite cell content in these same individuals. One of the aims was to determine if chronic skeletal muscle contraction in the form of regular endurance exercise performed over the adult lifespan would alter these two key components of skeletal muscle cellular regulation and adaptation and help explain our previous findings in men and women. An additional aim was to probe deeper into our previous slow and fast myofiber size findings by determining if LLE would alter the distribution of small and large myofibers in a fiber type or sex‐specific fashion. Our previous myofiber size measurements were completed in conjunction with time intensive physiological measurements (i.e., strength, speed, and power) on isolated (i.e., dissected) myofibers (Gries et al., 2019; Grosicki et al., 2021). Thus, the current myonuclear and satellite cell measurements, requiring the concurrent measurement of myofiber size in histological cross‐sections, allowed for a greater total number of myofibers to be investigated. This expanded our ability to explore a histogram analysis of the myofiber size in the studied cohorts.

## 2 METHODS

### 2.1 Subjects

Sixty‐eight subjects from the greater Muncie, IN area were recruited and enrolled in the study. Enrollment standards were stringent to include true lifelong exercisers with ~50 years of continuous exercise history and healthy age‐matched individuals with no apparent health problems or morbidities. All enrolled subjects were free of chronic illness (cardiac, pulmonary, liver, or kidney abnormalities, cancer, hypertension, diabetes, or other metabolic disorders), orthopedic limitations, and did not smoke or use any form of tobacco. Of the 451 people initially interviewed, 68 were accepted and included in this investigation (for more details on recruitment, see Gries et al. 2018). This study was approved by the Ball State University Institutional Review Board. Study procedures, risks, and benefits were explained to the subjects prior to obtaining their written consent to participate.

The final cohorts recruited included lifelong endurance exercisers (LLE: 21 men, seven women, ~75 years), age‐matched old healthy controls (OH: 10 men, 10 women, ~75 years), and young exercisers (YE: 10 men, 10 women, ~25 years). Detailed subject characteristics are provided in Table 1 and related methodologies (e.g., body composition, aerobic capacity, muscle size (MRI), muscle strength, and power) have been reported previously (Chambers et al., 2020; Gries et al., 2018). The LLE individuals recruited exercised on average ~ 5 days per week, ~7 h per week, for ~50 years. When analyzing all recruited LLE exercise histories, it became apparent that there were two subgroups within the LLE men, one training more intensely for competition and performance (LLE‐P, *n* = 14) and the other training primarily for fitness (LLE‐F, *n* = 7) (Gries et al., 2018). The LLE‐P group included several national caliber cyclists, a Race Across America record holder, a previous National Collegiate Athletic Association cross‐country All‐American, and many that continued to compete in endurance related events at a local, regional, and national level. Because of the small sample size (*n* = 7), LLE women were not subdivided and ranged from fitness to performance. The LLE women included several individuals who continue to compete in local, regional, and national races, including a national level Masters track and field athlete and one subject that cycled ~4000 miles a year at time of testing. The OH group consisted of healthy individuals that were not involved in any structured exercise training; however, participation in leisure activities (e.g., golfing, leisurely walking, and community service) was not grounds for exclusion. Young exercisers consisted of active individuals who exercised 4–6 day/wk for ~7 h/wk. Detailed exercise histories are presented in Table 2 (Gries et al., 2018).

**TABLE 1:** Subject characteristics.

				Men				
	Women			LLE				
	YE (*n* = 10)	LLE (*n* = 7)	OH (*n* = 10)	YE (*n* = 10)	Combined (*n* = 21)	Performance (*n* = 14)	Fitness (*n* = 7)	OH (*n* = 10)
Age, year	25 ± 2*	72 ± 4	75 ± 3	25 ± 1*	74 ± 4	74 ± 4	75 ± 4	75 ± 2
Height, cm	167 ± 6^†^	164 ± 6	157 ± 6	181 ± 6	180 ± 7	179 ± 7	182 ± 7	177 ± 7
Weight, kg	60 ± 7	61 ± 10	65 ± 4	75 ± 8	79 ± 9	77 ± 7	83 ± 12	88 ± 9*
BMI, kg·m^−2^	21 ± 2	23 ± 3	27 ± 2*	23 ± 2	24 ± 2	24 ± 2	25 ± 3	28 ± 1*
Body fat, %	23 ± 4*	30 ± 5*	41 ± 5*	18 ± 7*	24 ± 5*	22 ± 4^‡^	27 ± 4	32 ± 4*
VO_2_max, ml·kg^−1^·min^−1^	44 ± 8*	26 ± 5*	18 ± 3*	53 ± 8*	34 ± 6*	38 ± 4^‡^	27 ± 4	22 ± 4*
Quadriceps CSA, cm^2^	59 ± 6*	42 ± 5	40 ± 4	78 ± 9*	67 ± 7*	68 ± 6	65 ± 9	56 ± 8*
Quadriceps strength, Nm	138 ± 32*	105 ± 24	82 ± 13	210 ± 38*	165 ± 26*	166 ± 27	163 ± 27	134 ± 30*
Quadriceps 1RM, kg	80 ± 18*	54 ± 9	42 ± 6	121 ± 14*	86 ± 14	86 ± 10	85 ± 21	78 ± 13
Quadriceps power, W	404 ± 121*	221 ± 49	146 ± 33	699 ± 95*	370 ± 83	365 ± 45	377 ± 133	318 ± 133
Steps per day	11,518 ± 4441^†^	7463 ± 1807	6801 ± 2601	9404 ± 2007	9560 ± 2766	9369 ± 2713	10,006 ± 3098	5813 ± 1543*

*Note*: Data presented as mean ± SD. Data previously presented as part of our other reports on these cohorts (Gries et al., 2018; Chambers et al., 2020).

Abbreviations: %, percent; 1RM, one‐repetition maximum; cm, centimeters; CSA, cross‐sectional area; F, fitness; kg, kilograms; LLE, lifelong exercisers; m, meter; min, minute; ml, milliliters; Nm, newton‐meters; OH, old healthy; P, performance; W, watts; y, years; YE, young exercisers.

* *p* < 0.05 versus other groups.

^†^ *p* < 0.05 versus OH.

^‡^ *p* < 0.05 versus LLE‐F.

**TABLE 2:** Exercise training histories.

				Men				
	Women			LLE				
	YE (*n* = 10)	LLE (*n* = 7)	OH (*n* = 10)	YE (*n* = 10)	Combined (*n* = 21)	Performance (*n* = 14)	Fitness (*n* = 7)	OH (*n* = 10)
Total training, y	5 ± 1	48 ± 5	–	5 ± 1	53 ± 6	53 ± 5	53 ± 7	–
Competitive focus	–	–	–	Yes	–	Yes	No	–
Lifetime average								
Frequency, day·wk^−1^	–	4.6 ± 1.7	–	–	4.5 ± 1.6	4.4 ± 1.6	4.6 ± 1.8	–
Duration, h·wk^−1^	–	6.6 ± 3.6	–	–	7.3 ± 5.4	7.6 ± 5.5	6.6 ± 5.2	–
Intensity^a^	–	1.9 ± 0.7	–	–	2.0 ± 0.6	2.1 ± 0.6^b^	1.8 ± 0.6	–
Current decade								
Frequency, day·wk^−1^	5.4 ± 1.6	4.7 ± 1.1	–	5.1 ± 0.7	4.7 ± 1.4	4.5 ± 1.2	4.9 ± 1.8	–
Duration, h·wk^−1^	7.3 ± 3.4	6.8 ± 2.6	–	7.0 ± 2.2	8.1 ± 4.8	8.5 ± 4.8	7.4 ± 5.0	–
Intensity^a^	2.6 ± 0.4	2.1 ± 1.0	–	2.8 ± 0.3	2.0 ± 0.5	2.2 ± 0.3^b^	1.5 ± 0.5	–

*Note*: Data presented as mean ± SD. Data previously presented as part of our other reports on these cohorts (Gries et al., 2018; Chambers et al., 2020).

Abbreviations: F, fitness; h, hours; LLE, lifelong exercisers; OH, old healthy; P, performance; wk, weeks; y, years; YE, young exercisers.

^a^ Self‐reported intensity weighted averages: 1 (light), 2 (moderate), and 3 (hard).

^b^ *p* < 0.05 versus LLE‐F.

### 2.2 Skeletal muscle biopsies

Subjects were asked to refrain from structured exercise or physical activity for 72 h prior to the skeletal muscle biopsy. Subjects were asked to not consume anything but water following their regular evening meal the night before, and to arrive to the laboratory in a fasted state. Following arrival, subjects rested quietly in the supine position for 30 min before a muscle biopsy was taken from the vastus lateralis using a Bergström needle (Bergström, 1962). A portion of the skeletal muscle biopsy was cut and oriented longitudinally in mounting medium (Tragacanth, Sigma®, St. Louis, MO) atop a small cork, frozen in isopentane cooled in liquid nitrogen (LN_2_), and stored in LN_2_ until analysis.

### 2.3 Skeletal muscle sectioning

Skeletal muscle samples were sectioned using a microtome‐cryostat (HM 525; Microm, Walldorf, Germany) at −20°C. Tubes containing samples were taken from LN_2_ storage and placed within the microtome‐cryostat chamber. Following temperature equilibration, the samples were placed on a chuck (Microm International, Walldorf, Germany) and secured in place with Tissue‐Tek O.C.T. embedding medium (Sakura Finetek, Torrance, CA) and 10 μm serial sections were cut and placed on 25 × 75 × 1 mm frosted microscope slides (Fisherbrand, Pittsburg, PA). Three sections were placed on each of three slides for the following fluorescence stains: (1) fiber typing: MHC I and IIa, (2) fiber typing: MHC IIx, and (3) myonuclei and satellite cells. Slides were stored at −20°C overnight until the start of the staining protocols.

### 2.4 Skeletal muscle staining

#### 2.4.1 Fiber type, myonuclei, and satellite cells

Sections for fiber typing and myonuclear/satellite cell analyses were immunostained using a protocol based on those described by Bailly et al. (2020) and Murach et al. (2019), and further refined in our laboratory. Briefly, the morning following sectioning, slides were taken from −20°C and allowed to air dry at room temperature for 10 min. Sections were then fixed with pure acetone for 15 min. After the acetone dried from the slides, a hydrophobic pen (ImmEDGE Hydrophobic Barrier Pen, Vector Laboratories, Burlingame, CA) was used to encircle the skeletal muscle sections. When dry (~4 min), the slides were washed 3 × 5 min in 0.05% tween‐20 (Boston Bioproducts, Ashland, MA) in 1× TBS (Fisherbrand, Pittsburg, PA). Slides were carefully dried with a Kimwipe and light vacuum suction. Slides were placed in a humidified chamber and incubated with blocking solution (10% normal goat serum (Gibco, Waltham, MA), 2% bovine serum albumin (Thermo Scientific, Waltham, MA) in 1x TBS) for 1 h. Slides were dried as described above and then incubated with primary antibody cocktails for 1 h at room temperature. Slide #1 was incubated with fiber typing primary antibodies (mouse anti‐MHC I IgG2b (BA‐D5 concentrate, 1:200; Schiaffino, S., University of Padova) (Schiaffino et al., 1989), mouse anti‐MHC IIa IgG1 (SC‐71 concentrate, 1:150; Schiaffino, S., University of Padova) (Schiaffino et al., 1989), mouse anti‐laminin IgG2a (2E8 concentrate, 1:200; Engvall, E. S., La Jolla Cancer Research Foundation) (Schaart et al., 2004)). Slide #2 was incubated with MHC IIx specific primary antibodies (mouse anti‐MHC I and IIa IgG1 (BF‐35 concentrate, 1:200; Schiaffino, S., University of Padova) (Murach et al., 2019; Schiaffino et al., 1989), mouse anti‐laminin IgG2a (2E8 concentrate, 1:200); unstained was considered MHC IIx). Slide #3 was incubated with satellite cell primary antibodies (mouse anti‐PAX7 IgG1 (PAX7 concentrate, 1:25; Kawakami, A., Tokyo Institute of Technology) (Chal et al., 2015), mouse anti‐laminin IgG2a (2E8 concentrate, 1:200); mounted with DAPI mounting media to target myonuclei). Each group contained one section incubated only with blocking solution as a negative control. All primary monoclonal antibodies were obtaned from the Developmental Studies Hybridoma Bank, created by the NICHD of the NIH and maintained at The University of Iowa (Department of Biology, Iowa City, IA).

Following primary antibody incubation, slides were washed 4 × 5 min in 0.05% tween‐TBS, dried, and incubated with their respective secondary antibody cocktails in a humidified chamber in the dark for 1 h at room temperature (all secondary antibodies were obtained from Invitrogen, Waltham, MA). Slide #1 was incubated with goat anti‐mouse IgG2a Alexafluor (AF) 488 (lot # 2273777), 1:200; goat anti‐mouse IgG2b AF 555 (lot # 2184490), 1:200; and goat anti‐mouse IgG1 AF 647 (lot # 2185066), 1:200. Slide #2 was incubated with goat anti‐mouse IgG2a AF 488 (lot # 2273777), 1:200; and goat anti‐mouse IgG1 AF 555 (lot # 2273721), 1:200 (MHC IIx unstained). Slide #3 was incubated with goat anti‐mouse IgG2a AF 488 (lot # 2273777), 1:200; and goat anti‐mouse IgG1 AF 555 (lot # 2273721), 1:200.

Avoiding direct light in a dim room, slides were washed 3 × 5 min with 0.05% tween‐TBS, dried, and cover slips (Esco, Erie Scientific, Ramsen, MN) were used to cover sections using 1–2 drops of mounting media (slides #1 and 2) (ProLong Gold Antifade Mountant, Invitrogen, Waltham, MA) or 1–2 drops of mounting media with DAPI (slide # 3) (ProLong Gold Antifade Mountant with DAPI, Invitrogen, Waltham, MA).

### 2.5 Fluorescence imaging

Three sections were imaged per subject, two for fiber typing (MHC I and IIa, MHC IIx‐specific), and one for myonuclear and satellite cell content which also included cross‐sectional area measurements. All imaging took place in a dark imaging room. A microscope (BX‐51, 10×/0.30 objective lens, Olympus, Tokyo, Japan) with a fluorescence camera (DP26, Olympus, Tokyo, Japan) and full spectrum lamp (X‐Cite 120 PC, Excelitas Technologies, Waltham, MA) were used to capture whole‐section images across all applicable channels. L‐R parameters in cellSens entry imaging software (Olympus, Tokyo, Japan) were maintained constant within each section‐specific channel across all subject samples.

### 2.6 Image editing and analysis

Example images for the staining that was completed on the sections taken from the biopsies obtained from the men and women are presented in Figure S1, with supporting information provided in Tables [Link], [Link] (https://10.6084/m9.figshare.25718580). Additional specifics for each analysis are provided below.

Raw images were processed using FIJI (NIH, Bethesda, MD). For each section, all channels for each tile were merged (e.g., green, red, and blue), and then all merged images for a section were stitched together using the “Stitching” plug‐in feature on FIJI, forming a composite image of the whole section. This was done for all three different stain sets for all subjects.

To analyze serial section images in a fiber type specific manner, all three images for a subject were compared to ensure fibers aligned while being analyzed. To do so, fibers were numbered on the fiber typing (MHC I and MHC IIa) image on FIJI using the “Multi‐point” tool. It was required for all analogous fibers on other images to be intact (fully surrounded by laminin, not folded) for a myofiber to be counted. All usable fibers were assigned numbers which were used to systematically analyze all of the sections.

#### 2.6.1 Fiber typing and cross‐sectional area

To allow for fiber type specific myonuclei and satellite cells, fiber type for each numbered fiber was determined by color assessment of the MHC I (BA‐D5, 555 nm channel, red) and MHC IIa (SC‐71, 647 nm channel, pseudo‐colored blue) immunostained sections. Due to the cross‐reactivity of the SC‐71 MHC IIa antibody with MHC IIx protein (Murach et al., 2019), a secondary serial section immunostained with the BF‐35 antibody for MHC I and MHC IIa (555 nm channel, red) was analyzed to determine MHC IIa/MHC IIx hybrids (BF‐35 positive) and pure MHC IIx fibers (BF‐35 negative).

To allow for normalization of the myonuclei and satellite cell data, cross‐sectional area was measured on the myonuclear and satellite cell content images. To set the scale, the pixel distance across 100 μm on an image of a calibration ruler was measured at three different points and averaged; the scale was set to 2.9167 pixels per μm. A drawing tablet (WACOM, Saitama, Japan) was used to trace the perimeters of fibers on FIJI using the “Freehand Selections” tool. Cross‐sectional area was measured with the “Measure” function and color‐based fiber type was recorded for each fiber. The MHC IIx image was cross‐referenced to identify any pure MHC IIx fibers, which show up on this image as unstained (Murach et al., 2019). Fiber type distribution was determined as the number of myofibers identified for each fiber type (MHC I, MHC I/IIa, MHC IIa, MHC IIa/x, or MHC IIx) relative to the total number of fibers analyzed, and expressed as a percentage.

#### 2.6.2 Myonuclear and satellite cell content

To measure myonuclear and satellite cell content, images immunostained for Pax7 and mounted with DAPI were opened in FIJI. Using the “Cell Counter” feature, each fiber was analyzed for number of myonuclei and number of satellite cells. Myonuclei were counted if they were DAPI‐positive and within or on (but not crossing) the laminin boundary of each myofiber. Satellite cells were counted if DAPI‐positive nuclei were also Pax7‐positive (red) and within or on the basal lamina of the myofiber. Satellite cells were excluded from myonuclear counts. Each was labeled with an appropriate marker representing the target (myonucleus or satellite cell) and fiber type it was associated with (MHC I, MHC I/IIa, MHC IIa, MHC IIa/x, or MHC IIx). Total counts were recorded for each fiber.

### 2.7 Statistical analyses

Comparisons of myonuclei/fiber, myonuclear domain, and satellite cells/fiber in MHC I and MHC IIa fibers among YE, LLE, and OH were completed using one‐way ANOVAs. Hybrid (MHC I/IIa, MHC IIa/IIx) and pure MHC IIx fibers were not statistically analyzed due to the low representation of these fiber types. Intra‐sex comparisons were completed because of the relatively small number of LLE women included in the investigation (due to the much smaller pool of available women in this group because of the social norms at the time of the exercise boom and the difference in sports and exercise opportunities afforded women compared with men). This same approach has been taken in our other publications on these same cohorts of men and women (Chambers et al., 2020; Gries et al., 2018, 2019; Grosicki et al., 2021; Lavin et al., 2020a, 2020b; Perkins et al., 2020, 2024; Raue et al., 2023). Post hoc comparisons were completed with Tukey's test. LLE‐P and LLE‐F were compared using an independent two‐tailed *T*‐test with Levene's Test for equality of variance. Significance was accepted at *p* ≤ 0.05, and trends reported when *p* > 0.05 and ≤0.1. Histograms were created for each group to present the frequency distribution of myofiber sizes in 500 μm^2^ bins, and further summarized as groupings of these bins. The histograms intrinsically provide the comparisons within and across groups. Separate from the histograms, all data are reported as mean ± SD.

## 3 RESULTS

Table S4 provides the number of myofibers studied for each measurement based on group and fiber type for the men and women (https://10.6084/m9.figshare.25718580). Tables S5 and S6 provide the mean (±SD) data of all the groups for fiber type and fiber size that were collected while generating the data for the main outcome variables presented below (https://10.6084/m9.figshare.25718580).

### 3.1 Myonuclear content and domain

**FIGURE 1:**
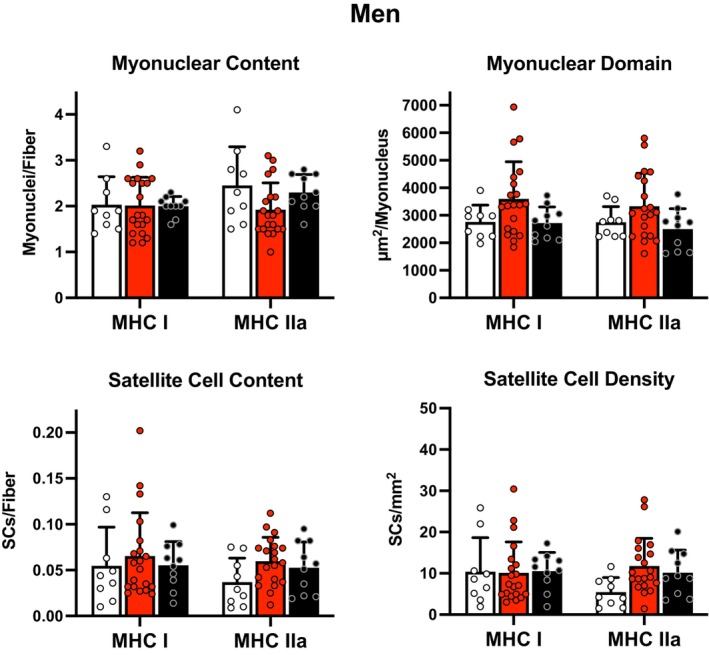
Fiber type specific myonuclear content, myonuclear domain, satellite cell content, and satellite cell density in men. LLE, lifelong excercisers; MHC, myosin heavy chain; OH, old healthy; YE, young exercisers; μm^2^, micrometers squared. There was a trend (*p* = 0.07) for LLE to have a greater satellite cell density in MHC IIa fibers compared to YE. Data are mean ± SD, along with individual data.

**FIGURE 2:**
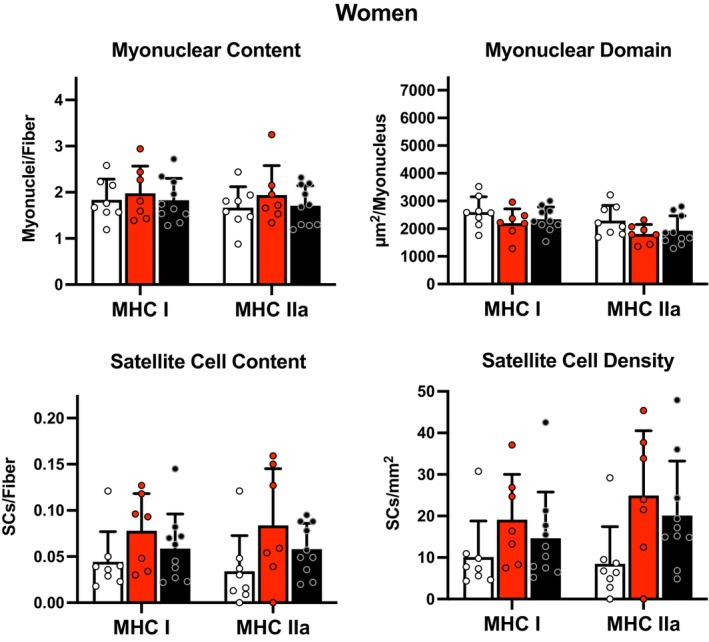
Fiber type specific myonuclear content, myonuclear domain, satellite cell content, and satellite cell density in women. LLE, lifelong excercisers; MHC, myosin heavy chain; OH, old healthy; YE, young exercisers; μm^2^, micrometers squared. There was a trend (*p* = 0.06) for LLE to have a greater satellite cell density in MHC IIa fibers compared to YE. Data are mean ± SD, along with individual data.

There were no differences in fiber type specific myonuclear content or myonuclear domain across the three cohorts of men (*p* > 0.05, Figure 1). There were no differences in fiber type specific myonuclear content or myonuclear domain across the three cohorts of women (*p* > 0.05, Figure 2).

### 3.2 Satellite cell content and density

There were no differences in fiber type specific satellite cell content or satellite cell density across the three cohorts of men (*p* > 0.05, Figure 1). However, there was a trend (*p* = 0.07) for LLE to have a greater satellite cell density in MHC IIa fibers compared to YE. There were no differences in fiber type specific satellite cell content across the three cohorts of women (*p* > 0.05, Figure 2). However, there was a trend (*p* = 0.06) for LLE to have a greater satellite cell density in MHC IIa fibers compared to YE. Of note, two women (one YE and one LLE) had limited to no measurable satellite cells in their MHC IIa fibers, which was likely related to the low (10%–13%) MHC IIa fibers in their samples (Mackey et al., 2009).

### 3.3 Myofiber size distribution

**FIGURE 3:**
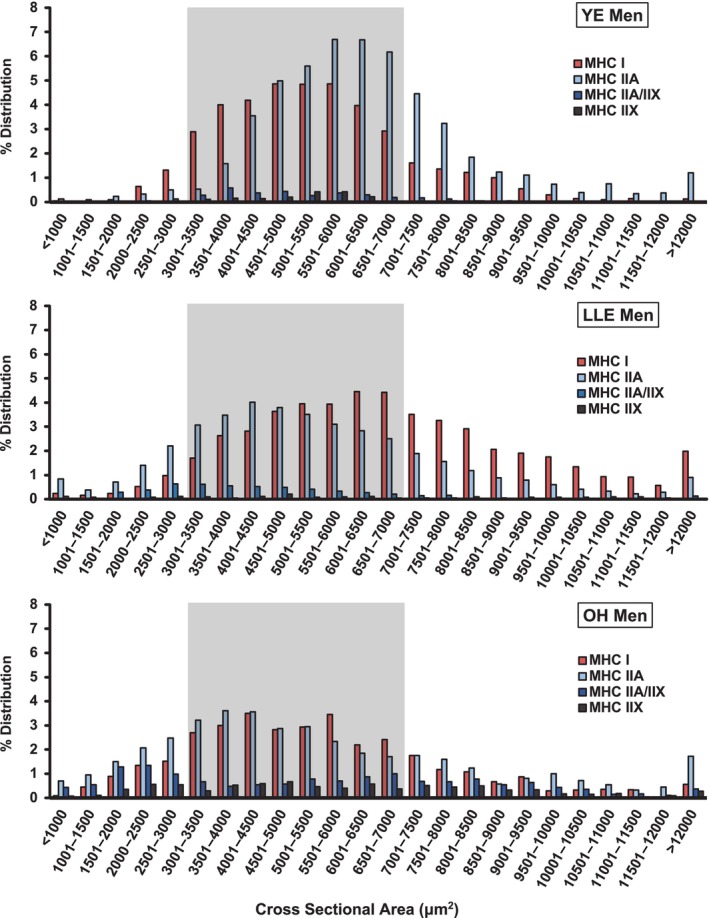
Skeletal muscle fiber type specific cross‐sectional area distribution in YE, LLE, and OH men. Gray shading highlights fibers 3000–7000 μm^2^. Fibers <3000 μm^2^ were considered “small” and fibers >7000 μm^2^ were considered “large”. LLE, lifelong excercisers; MHC, myosin heavy chain; OH, old healthy; YE, young exercisers; μm^2^, micrometers squared.

**FIGURE 4:**
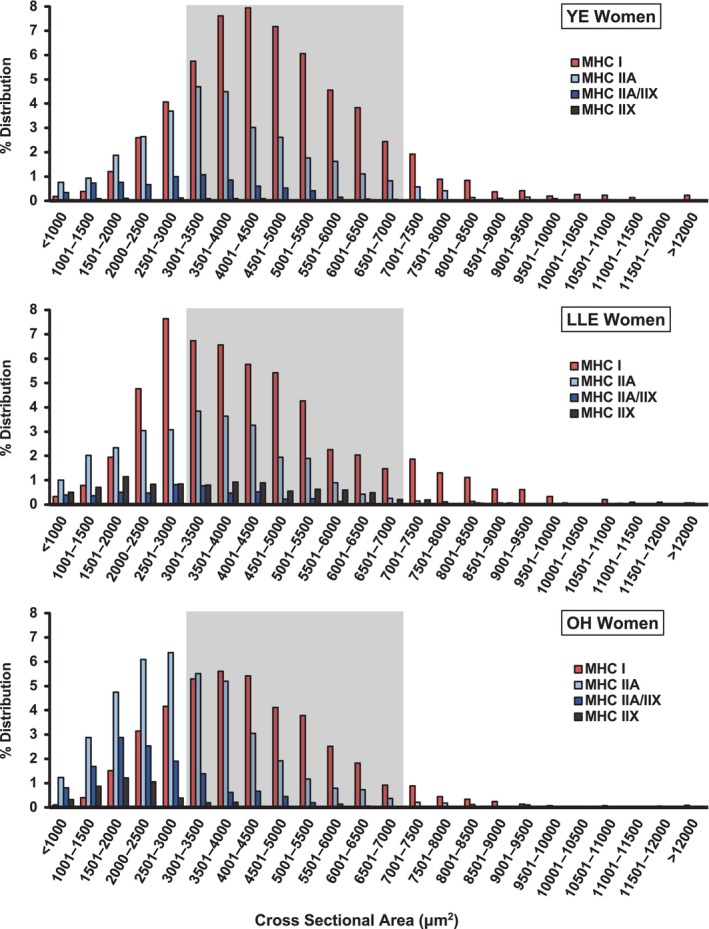
Skeletal muscle fiber type specific cross‐sectional area distribution in YE, LLE, and OH women. Gray shading highlights fibers 3000–7000 μm^2^. Fibers <3000 μm^2^ were considered “small” and fibers >7000 μm^2^ were considered “large”. LLE, lifelong exercisers; MHC, myosin heavy chain; OH, old healthy; YE, young exercisers.

Fiber type specific cross‐sectional area distributions were plotted and fibers <3000 μm^2^, 3000–7000 μm^2^, and >7000 μm^2^ were compared across groups (Figures 3 and 4, Table 3). These groupings and designations (i.e., small, normal, or large) were chosen by us based on our experience with myofiber size measurements across a wide range of populations. There are no set normative data on myofiber size, to our knowledge, in the literature.

**TABLE 3:** Myofiber size (μm^2^) histogram summary of different myosin heavy chain (MHC) types.

Group		I	IIa	IIa/IIx	IIx	Total II	Group Total
Men							
<3000 (Small)	YE	2	1	0	0	2	4
	LLE	2	6	1	0	7	10
	OH	4	8	5	2	14	18
3000–7000	YE	33	36	3	2	40	73
	LLE	28	26	3	1	31	59
	OH	23	22	6	4	32	55
>7000 (Large)	YE	7	16	0	0	16	23
	LLE	21	9	1	0	10	32
	OH	7	11	5	3	19	26
Men LLE subgroups							
<3000 (Small)	LLE‐P	2	4	2	0	6	8
	LLE‐F	3	9	1	0	11	14
3000–7000	LLE‐P	29	32	3	0	35	64
	LLE‐F	25	16	4	1	21	48
>7000 (Large)	LLE‐P	19	9	0	0	9	28
	LLE‐F	25	10	3	0	13	39
Women							
<3000 (Small)	YE	8	10	3	0	14	22
	LLE	15	11	3	4	18	34
	OH	9	21	10	4	35	45
3000–7000	YE	45	20	4	0	24	70
	LLE	35	16	2	5	24	58
	OH	29	19	3	0	23	52
>7000 (Large)	YE	5	2	0	0	2	7
	LLE	6	1	0	0	1	8
	OH	2	1	0	0	1	3

*Note*: Data are % frequency distribution. Total II is the sum of MHC IIa, IIa/IIx, and IIx. Small differences in the individual numbers added up and the Total II or Group Total is related to rounding (e.g., MHC types with <0.5% are listed as 0). No I/IIa data are presented as the amount was negligible in all groups. See Figures 3, 4, 5 for full histograms of these data.

Abbreviations: F, fitness; LLE, lifelong exercisers; OH, old healthy; P, performance; YE, young exercisers.

#### 3.3.1 Men

YE men (73%) had a higher proportion of muscle fibers in the 3000–7000 μm^2^ range compared with both LLE men (59%) and OH men (55%). However, LLE had a higher proportion of large fibers (>7000 μm^2^) compared with YE and OH (Table 3), primarily due to having three times higher proportion of large MHC I fibers (LLE: 21%, YE: 7%, OH: 7%). In addition, OH (18%) had more than four times the proportion of small fibers (<3000 μm^2^) compared to YE (4%) and nearly double compared with LLE (10%). This was primarily due to the higher proportion of small fibers containing MHC II (OH: 14%, YE: 2%, LLE: 7%).

#### 3.3.2 Women

YE women (70%) had a higher proportion of muscle fibers in the 3000–7000 μm^2^ range compared with both LLE women (58%) and OH women (52%). However, all three groups had relatively few large fibers (YE: 7%, LLE: 8%, OH: 3%), with the balance categorized as small fibers (Table 3). OH had a relatively high proportion of small fibers (45%), which was more than double YE (22%) and primarily due to a higher proportion of small fibers containing MHC II (OH: 35%, YE: 14%). Interestingly, LLE maintained the proportion of small fibers containing MHC II (18%) similar to YE; however, their proportion of small MHC I fibers (15%) was nearly double YE (8%) and OH (9%).

### 3.4 LLE men: Performance versus Fitness

**FIGURE 5:**
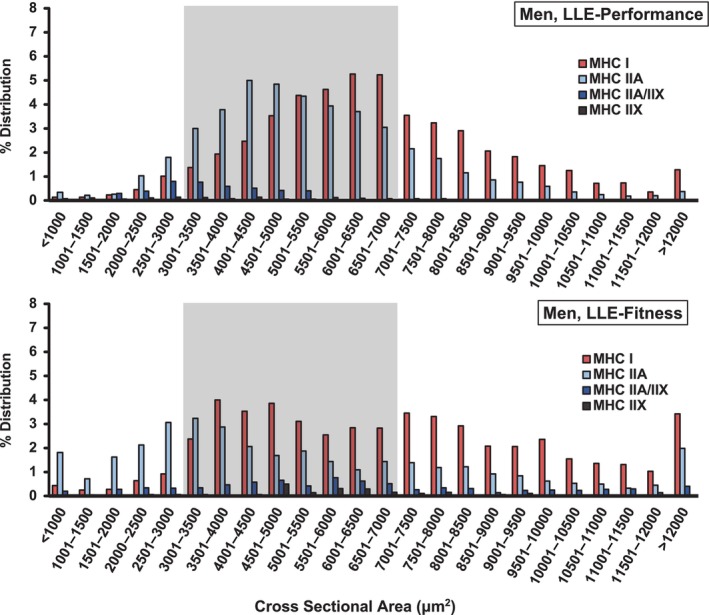
Skeletal muscle fiber type specific cross‐sectional area distribution in LLE‐P and LLE‐F men. Gray shading highlights fibers 3000–7000 μm^2^. Fibers <3000 μm^2^ were considered “small” and fibers >7000 μm^2^ were considered “large”. LLE‐F, lifelong exercisers‐fitness; LLE‐P, lifelong exercisers‐performance; MHC, myosin heavy chain; μm^2^, micrometers squared.

Fiber type specific myonuclear content, myonuclear domain, satellite cell content, and satellite cell density did not differ between LLE‐P and LLE‐F (*p* > 0.05). LLE‐P (64%) had a higher proportion of muscle fibers in the 3000–7000 μm^2^ range compared with LLE‐F (48%) (Figure 5, Table 3). This difference was driven by LLE‐F having a higher proportion of large (LLE‐P: 28%, LLE‐F: 39%) and small (LLE‐P: 8%, LLE‐F: 14%) muscle fibers. The higher proportion of large fibers was driven by a higher proportion of both MHC I and MHC II containing fibers, while the higher proportion of small fibers was driven primarily by a higher proportion of MHC II containing fibers (Table 3).

## 4 DISCUSSION

We had the unique opportunity to examine slow and fast muscle fiber characteristics in men and women who had been consistent endurance exercisers for fitness and competitive purposes for more than 50 years. Their exercise profiles provide one of the more realistic “extremes” to examine the likelihood of myonuclei or satellite cell content being influenced by endurance exercise training over the lifespan. It is noteworthy that the exercise time commitment of the LLE groups was only ~4% of the available weekly minutes, which is less than the average time invested in television watching or social media in the United States (Dixson, 2023; Freeman, 2023). Overall, the main findings from this investigation indicated that (1) the myonuclear and satellite cell profile in skeletal muscle is relatively refractory to lifelong endurance exercise, and (2) the relative abundance of small and large slow and fast myofibers is influenced by lifelong endurance exercise, which is in turn different between men and women.

The lack of influence of lifelong endurance exercise training on the number of myonuclei per fiber compared with age‐matched non‐exercising counterparts in the septuagenarian LLE men and women in the current study is in agreement with other investigations of lifelong endurance exercising sexagenarian (Karlsen et al., 2015; Mackey et al., 2014) and octogenarian (Skoglund et al., 2023) men. Collectively, these data would suggest a preservation of myonuclear content across this three‐decade age span (~60–92 years) in elderly men and women, although more data are needed on women to confirm this interpretation. The similarity of myonuclear content compared to the young exercising men and women in the current study is also in agreement with the study of the sexagenarian men (Karlsen et al., 2015; Mackey et al., 2014), suggesting this was not influenced by the additional decade of aging combined with the higher amount of endurance exercise (~7 h vs. ~4 h) per week.

The myonuclear domain findings generally followed the myonuclear number findings with similar profiles reported for the slow and fast muscle fibers of the men and women (Figures 1 and 2). The ~30% higher (albeit nonsignificant) myonuclear domain in the slow fibers of the LLE men (Figure 1) does align with the larger (~25%–30%) slow fibers reported here (Table S6) and previously by us for this group (Grosicki et al., 2021). However, the myonuclear domain did not seem to be influenced in the generally smaller (~20%) fast fibers of the LLE and OH cohorts of men and women (Gries et al., 2019; Grosicki et al., 2021). The current findings are supported by the fiber type specific myonuclear profiles reported on the lifelong sexagenarian runners (Karlsen et al., 2015; Mackey et al., 2014) and further support the idea that the myonuclear domain may not be as tightly regulated as previously suggested (Murach et al., 2018). When interpreting the lifelong exercise data, it is likely that the regulation of the interplay between aging and endurance exercise over 50 years involves a different construct with respect to the myonuclei and control of muscle protein synthesis/degradation than has been considered with short‐term training studies focused on the ability to grow muscle fibers in aging individuals (Blocquiaux et al., 2020; Franco et al., 2019; Hikida et al., 1998; Kadi et al., 2004; Karlsen et al., 2019, 2020; Leenders et al., 2013; Lindström & Thornell, 2009; Mackey et al., 2007, 2011; Murach et al., 2021; Olsen et al., 2006; Petrella et al., 2006, 2008; Roth et al., 2001; Snijders et al., 2016, 2017, 2020; Thornell et al., 2003; Verdijk et al., 2009, 2014). The subjects in the current investigation were studied in a period that would be considered “steady state” with a prolonged, constant exercise stimulus provided to the muscle leading into obtaining the muscle biopsies. Furthermore, recent evidence shows that myonuclei from young runners and septuagenarian cyclists have more spherical, less deformable, and thicker nuclear lamina than age‐matched untrained individuals suggesting that exercise results in healthier myonuclei that could be important in muscle regulatory control and remodeling throughout the lifespan (Battey et al., 2023). Indeed, the ability of the myonuclei to provide adequate control over the production of the enzymes of aerobic energy metabolism and the components of capillarity appears to be maintained in to the eighth (Gries et al., 2018), ninth (Trappe et al., 2013), and tenth (Skoglund et al., 2023) decade in lifelong endurance exercisers.

The satellite cell number and density data from the three male cohorts from the current study generally align with the previous studies of endurance trained sexagenarian and octogenarian men (Mackey et al., 2014; Skoglund et al., 2023). As with the myonuclear data, these findings would suggest a preservation of satellite cell abundance across this three‐decade age span (~60–92 years) in elderly men, that is uninfluenced by lifelong endurance exercise. Our current findings from the women extend this trend to septuagenarian LLE women. Overall, it appears satellite cell abundance per se does not play a significant role in muscle mass regulation with aging or LLE. However, this does not preclude other regulatory involvement of satellite cells (or myonuclei) over the lifespan that is not considered with our current study design (Karlsen et al., 2020; Soendenbroe et al., 2022).

The fiber type specific myofiber size histogram data (Figures 3, 4, 5, Table 3) provide an expansion of our previous reports on these cohorts (Gries et al., 2019; Grosicki et al., 2021) to include a substantially larger number of muscle fibers (Table S4). Examination of the size distribution data shows that both YE men and women had ~70%–75% of their fibers in the range we characterized as “normal” (3000–7000 μm^2^). However, LLE in both the men and women was not able to prevent a substantial drop in the proportion of fibers in this range (58%–59%). In general (considering all three groups), the women had most of their fibers outside the “normal” range in the small (<3000 μm^2^) category, while the men had most of their fibers outside the “normal” range in the large (>7000 μm^2^) category. Congruent with the LLE men having larger slow (MHC I) fibers ((Grosicki et al., 2021), Table S6), they had triple the proportion of large (>7000 μm^2^) slow fibers compared to the other groups (LLE: 21%, YE: 7%, OH: 7%). Conversely, the LLE women had nearly double the proportion of small slow fibers compared with the YE and OH women. This contrasting slow fiber size response of the LLE women and men is interesting, but without any obvious explanation. However, LLE in both the aging women (OH: 35%, LLE: 18%) and men (OH: 14%, LLE: 7%) reduced the proportion of small fibers containing the fast MHC II isoforms by ~50%. Overall, the stimulus of LLE superimposed on the aging process appears to provide a divergent cellular response with respect to myofiber size in a fiber type‐ and sex‐specific fashion. It would be interesting to know the total muscle fiber numbers in the vastus lateralis (the biopsied muscle) for each group to be able to further interpret the current data, but in vivo technologies are not currently available to determine this in humans.

Differential responses in some physiological and skeletal muscle attributes due to a different training intensity over the lifespan in the LLE‐P versus LLE‐F men have previously been reported (Chambers et al., 2020; Gries et al., 2018, 2019; Grosicki et al., 2021; Lavin et al., 2020a, 2020b; Perkins et al., 2020). In the current investigation, training intensity did not seem to influence the myonuclei or satellite cell profiles, which is not surprising considering the other findings reported here for these variables. However, an increased training intensity (i.e., LLE‐P) did appear to compress the range of fiber size (i.e., reduce fiber size heterogeneity) across the fiber type spectrum compared with LLE‐F (Figure 5, Table 3). The small, normal, and large fiber size profiles of the LLE‐P (8%, 64%, and 28%) were much closer to the YE men (4%, 73%, and 23%) than LLE‐F (14%, 48%, and 39%), which in turn were closer to the OH men (18%, 55%, and 26%). Although speculative, this may be due to the muscle adapting to a more specific demand placed on it with the training of the performance oriented lifelong exercising men.

Lastly, we recognize the myocellular responses to aging and lifelong exercise are regulated by aspects other than the content of satellite cells (or myonuclei) (Dumont et al., 2015; Franco et al., 2019; Larsson et al., 2019; Mauro, 1961; McCarthy et al., 2011; Murach et al., 2021; Snijders et al., 2015; Soendenbroe et al., 2022; Thornell et al., 2003). Individual differences at the genetic level, as well as cellular control at the transcriptional and translational level regulate skeletal muscle adaptations (Adams & Bamman, 2012; Dennis et al., 2004; Lavin et al., 2019, 2022). Indeed, fast and slow muscle fiber transcriptome dynamics in response to exercise have been shown to differ among a subset of the YE, LLE, and OH men studied in the current investigation (Raue et al., 2023). Hormonal factors, acting at the endocrine, paracrine, and autocrine level, along with nutritional interactions also should be factored into the adaptive control mechanisms (Chow et al., 2022; Nunes et al., 2022; Rennie & Tipton, 2000; Severinsen & Pedersen, 2020). Additionally, it would be informative to examine the influence of lifelong resistance exercise (Toien et al., 2023) on skeletal muscle health, including the satellite cell profiles which have been shown to be responsive to resistance exercise in numerous, but not all studies (e.g., (Blocquiaux et al., 2020; Franco et al., 2019; Hikida et al., 1998; Kadi et al., 2004; Karlsen et al., 2019; Karlsen et al., 2020; Leenders et al., 2013; Lindström & Thornell, 2009; Mackey et al., 2007; Mackey et al., 2011; Murach et al., 2021; Olsen et al., 2006; Petrella et al., 2006; Petrella et al., 2008; Roth et al., 2001; Snijders et al., 2016; Snijders et al., 2017; Snijders et al., 2020; Thornell et al., 2003; Verdijk et al., 2009; Verdijk et al., 2014)). Interestingly, Soendenbroe et al. (2022) reported that men (68–82 years) that had been recreationally involved in “multiple sports through their adult life…that would lead to recruitment of type II myofibers in the lower extremities (high force or high speed)” had a higher number of satellite cells in their fast (type II) muscle fibers compared with a similarly aged sedentary group of men. Based on our experience with lifelong exercise studies (Gries et al., 2018; Trappe et al., 1995, 2013) and other reports in the literature (Toien et al., 2023) the potentially available cohort of septuagenarian (or older) lifelong resistance exercisers, especially women, is relatively small and will require some additional time for this cohort to develop.

In summary, the current study expands the limited knowledge base regarding skeletal muscle adaptations to a lifetime of regular endurance exercise in women and men. Collectively, the current study when combined with the previously available data on lifelong endurance exercising men (Mackey et al., 2014; Skoglund et al., 2023), generally support relatively stable myonuclei and satellite cell profiles across three decades (age 60–80's) with various endurance exercise modes (running, cycling, and cross‐country skiing), a range in weekly exercise volume (4–8 h/wk) and exercise intensity (moderate to vigorous). Overall, this endurance exercise dose is a relatively modest time investment (3%–5% of weekly time). The data presented here on women, along with our previous reports (Chambers et al., 2020; Gries et al., 2018, 2019; Lavin et al., 2020b), expand the even more limited data regarding lifelong exercising women and suggest similar findings as observed in the men; however, the smaller sample size and limited elderly age groups suggests this area requires more research. The fast and slow myofiber size distribution data add to our previous reports on whole muscle and myofiber size (Chambers et al., 2020; Gries et al., 2019; Grosicki et al., 2021) and suggest additional cellular regulation of muscle mass that is influenced by LLE, but differently between men and women. With additional time, more lifelong exercising individuals will be available to study and provide more insight into the effects of a physically active lifestyle on the aging process. This should include other modes of exercise, such as resistance exercise and combined endurance and resistance exercise training, that will improve our knowledge to provide guidance to men and women to enhance the quality of life throughout the aging process.

## AUTHOR CONTRIBUTIONS

CFM, KM, UR, TT, and ST conceived and designed research. CFM, CS, DJK, AG, KM, TT, and ST analyzed data. CFM, CS, TT, and ST interpreted results of experiments. CFM, TT, and ST prepared figures and drafted manuscript. All authors performed experiments, edited and revised manuscript, and approved final version of manuscript.

## FUNDING INFORMATION

This research was supported by the National Institutes of Health Grant R01‐AG038576 and the Ball State University Human Bioenergetics Program.

## CONFLICT OF INTEREST STATEMENT

No conflicts of interest, financial or otherwise, are declared by the authors.

## Supporting information


Figure S1.

Table S1.

Table S2.

Table S3.

Table S4.

Table S5.

Table S6.

## Data Availability

Data will be made available by the authors upon reasonable request. Related data are also available at https://doi.org/10.6084/m9.figshare.25718580.
