# Supplementary material for: Fast and slow myofiber nuclei, satellite cells, and size distribution with lifelong endurance exercise in men and women
Source: Physiol Rep. 2024 Jul 10;12(13):e16052. doi: 10.14814/phy2.16052 (PMC11236482; doi:10.14814/phy2.16052)
Supplement: Supplementary file 1 — Figure S1. [file PHY2-12-e16052-s002.pdf]

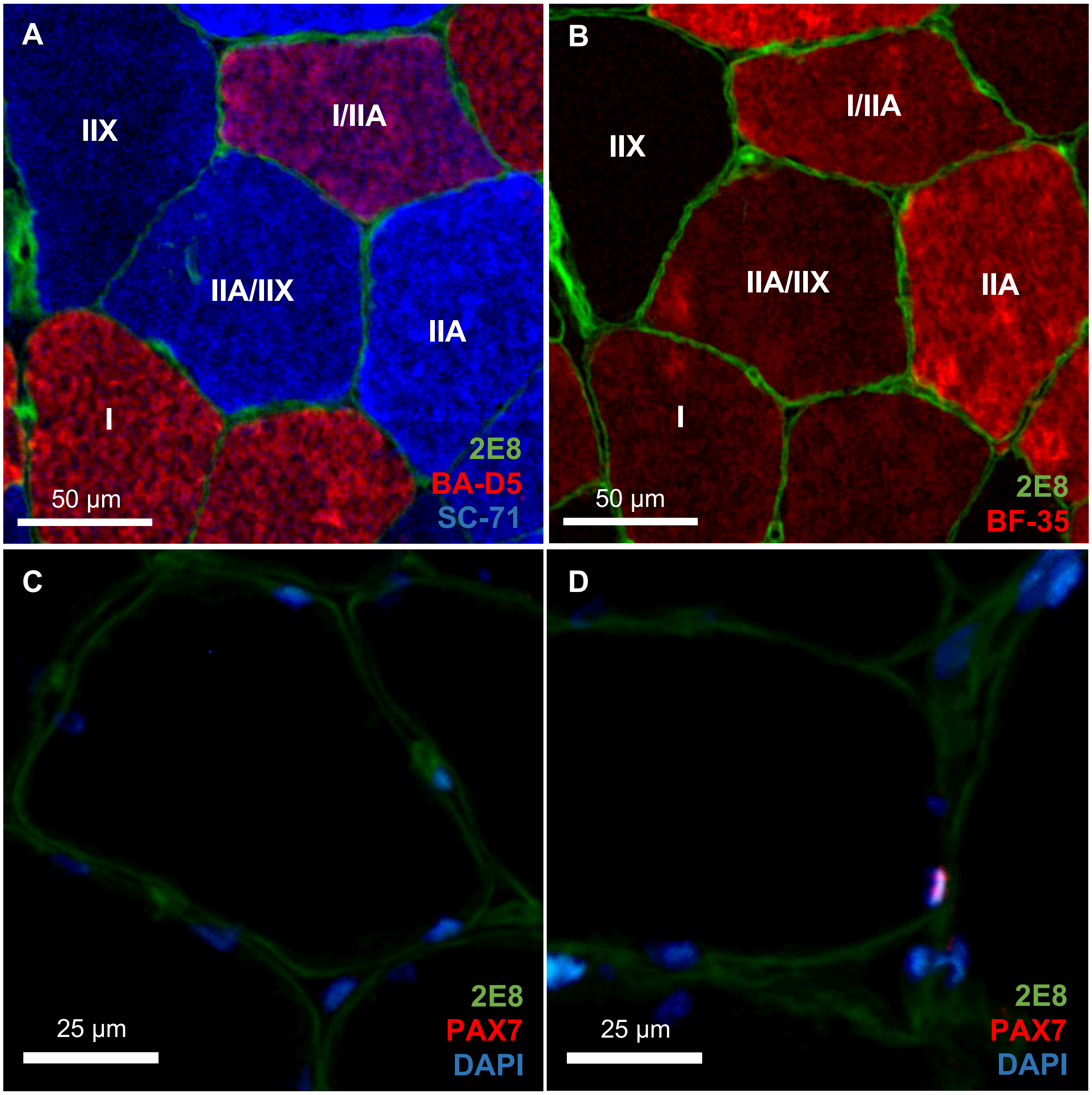

Figure S1. (A) Skeletal muscle immunofluorescence staining for MHC I (BA-D5) and MHC IIA (SC-71) fibers. (B) Immunofluorescence staining for pure MHC IIX fibers, identified as BF-35 negative. (C-D) Myonuclear (DAPI) and satellite cell (DAPI and PAX7 colocalization) immunofluorescence staining. A-D were counterstained with 2E8 for laminin.
