# Supplementary material for: Fast and slow myofiber nuclei, satellite cells, and size distribution with lifelong endurance exercise in men and women
Source: Physiol Rep. 2024 Jul 10;12(13):e16052. doi: 10.14814/phy2.16052 (PMC11236482; doi:10.14814/phy2.16052)
Supplement: Supplementary file 2 — Table S1. [file PHY2-12-e16052-s005.docx]

| **Table S1.** Microscope camera settings for fluorescence imaging. | | | | | | | |
| --- | --- | --- | --- | --- | --- | --- | --- |
| **Cellular Target** | **Filter** | | | **Lamp Intensity (%)** | **L** | | **R** |
| *Fiber Typing* | | | | | | | |
| Laminin (AF488) | | 488-nm | 50 | | | 10 | 80 |
| MHC I, BA-D5 (AF555) | | 555-nm | 25 | | | 3 | 80 |
| MHC IIa, SC-71 (AF647) | | 647-nm | 50 | | | 0 | 90 |
| MHC IIx, BF-35 (AF555) | | 555-nm | 12 | | | 6 | 80 |
| *Myonuclei and Satellite Cells* | | | | | | | |
| Nuclei (DAPI) | | 350-nm | 25 | | | 30 | 150 |
| Laminin, 2E8 (AF488) | | 488-nm | 50 | | | 10 | 80 |
| Satellite Cells, PAX7 (AF555) | | 555-nm | 100 | | | 3 | 50 |

%, percent; L, left; R, right; AF, AlexaFluor; DAPI, 4′,6-diamidino-2-phenylindole.
