# Supplementary material for: Fast and slow myofiber nuclei, satellite cells, and size distribution with lifelong endurance exercise in men and women
Source: Physiol Rep. 2024 Jul 10;12(13):e16052. doi: 10.14814/phy2.16052 (PMC11236482; doi:10.14814/phy2.16052)
Supplement: Supplementary file 4 — Table S3. [file PHY2-12-e16052-s004.docx]

| **Table S3.** Skeletal muscle fiber typing antibody reactivity. | | | |
| --- | --- | --- | --- |
| **Fiber Type** | **Antibody Set 1** | | **Antibody Set 2** |
|  | **BA-D5 (Red)** | **SC-71 (Blue)** | **BF-35 (Red)** |
| *Pure Fibers* | | | |
| **MHC I** | **+** | **-** | **+ (Weak)** |
| **MHC IIa** | **-** | **+** | **+ (Strong)** |
| **MHC IIx** | **-** | **+ (Weak)** | **-** |
| *Hybrid Fibers* | | | |
| **MHC I/IIa** | **+** | **+** | **+** |
| **MHC IIa/IIx** | **-** | **+** | **+** |

MHC, myosin heavy chain; +, positive staining; -, unstained.
