# Supplementary material for: Fast and slow myofiber nuclei, satellite cells, and size distribution with lifelong endurance exercise in men and women
Source: Physiol Rep. 2024 Jul 10;12(13):e16052. doi: 10.14814/phy2.16052 (PMC11236482; doi:10.14814/phy2.16052)
Supplement: Supplementary file 5 — Table S4. [file PHY2-12-e16052-s007.docx]

**Table S4.** Number of subject muscle fibers analyzed for fiber type distribution,

cross-sectional area, and myonuclear and satellite cell content.

| **Group** | **Fiber Type** | | **Cross Sectional Area** | | **Myonuclei and SCs** | |
| --- | --- | --- | --- | --- | --- | --- |
|  | **Mean** | **Total** | **Mean** | **Total** | **Mean** | **Total** |
| **Women** | **683±220** | **17078** | **683±220** | **17078** | **683±220** | **17078** |
| YE | 657±181 | 5258 | 657±181 | 5258 | 657±181 | 5258 |
| LLE | 606±167 | 4239 | 606±167 | 4239 | 606±167 | 4239 |
| OH | 758±271 | 7581 | 758±271 | 7581 | 758±271 | 7581 |
|  |  |  |  |  |  |  |
| **Men** | **558±248** | **22311** | **558±248** | **22311** | **560±251** | **21848** |
| YE | 521±180 | 4693 | 521±180 | 4693 | 521±180 | 4693 |
| LLE | 535±163 | 11235 | 535±163 | 11235 | 539±167 | 10772 |
| LLE-P | 553±132 | 7748 | 553±132 | 7748 | 560±135 | 7285 |
| LLE-F | 498±221 | 3487 | 498±221 | 3487 | 498±221 | 3487 |
| OH | 638±411 | 6383 | 638±411 | 6383 | 638±411 | 6383 |
|  |  |  |  |  |  |  |
| **All** | **606±244** | **39389** | **606±244** | **39389** | **608±245** | **38926** |

Mean data provided as mean±SD. SCs, satellite cells; YE, young exercisers; LLE, lifelong exercisers;

OH, old healthy; P, performance; F, fitness.
