# Supplementary material for: Fast and slow myofiber nuclei, satellite cells, and size distribution with lifelong endurance exercise in men and women
Source: Physiol Rep. 2024 Jul 10;12(13):e16052. doi: 10.14814/phy2.16052 (PMC11236482; doi:10.14814/phy2.16052)
Supplement: Supplementary file 6 — Table S5. [file PHY2-12-e16052-s001.docx]

| **Table S5.** Skeletal muscle fiber type distribution. | | | | | |
| --- | --- | --- | --- | --- | --- |
|  | **Fiber Type Distribution** | | | | |
|  | **MHC I** | **MHC I/IIa** | **MHC IIa** | **MHC IIa/IIx** | **MHC IIx** |
|  | *Women* | | | | |
| **YE** | 59.3±15.0^†^ | 1.1±1.9 | 31.6±10.7 | 7.3±6.9 | 0.7±0.9 |
| **LLE** | 56.3±16.9^a^ | 0.7±1.4 | 28.2±16.3 | 5.1±5.6^a^ | 9.6±16.7 |
| **OH** | 41.2±10.7 | 0.4±0.4 | 40.8±12.9 | 13.4±9.0 | 4.3±5.3 |
|  | *Men* | | | | |
| **YE** | 41.3±10.3 | 0.5±0.5 | 52.8±9.0^c^ | 3.5±5.1 | 1.9±4.8^b^ |
| **LLE**  LLE-P  LLE-F | 50.8±17.5^†^  50.1±15.3  52.4±22.6 | 1.1±1.9  0.5±0.7  2.3±3.0^d^ | 40.9±14.9  43.9±15.3  35.0±13.0 | 6.0±7.4  4.8±3.8  8.3±11.9 | 1.2±2.9^†^  0.8±1.7  2.0±4.6 |
| **OH** | 34.8±14.0 | 1.0±1.6 | 40.6±13.7 | 15.1±11.7* | 8.5±10.7 |
| Data presented as mean±SD. Numbers reflect the % calculated from myofiber numbers. YE, young exercisers; LLE, lifelong exercisers; OH, old healthy; MHC, myosin heavy chain. **P*<0.05 vs. other groups; ^†^*P*<0.05 vs. OH; ^a^*P*=0.09 vs. OH; ^b^*P*=0.06 vs. OH; ^c^*P*=0.08 vs. LLE, ^d^*P*<0.05 vs. LLE-P. | | | | | |
