# Supplementary material for: Fast and slow myofiber nuclei, satellite cells, and size distribution with lifelong endurance exercise in men and women
Source: Physiol Rep. 2024 Jul 10;12(13):e16052. doi: 10.14814/phy2.16052 (PMC11236482; doi:10.14814/phy2.16052)
Supplement: Supplementary file 7 — Table S6. [file PHY2-12-e16052-s003.docx]

**Table S6.** Fiber type specific cross-sectional area.

|  | **MHC I** | **MHC I/IIa** | **MHC IIa** | **MHC IIa/IIx** | **MHC IIx** |
| --- | --- | --- | --- | --- | --- |
|  | *Women* | | | | |
| **YE** | 4649±979 | 3871±1519 | 3722±975 | 2866±1239 | 2280±1133 |
| **LLE** | 4271±1446 | 2787±1256 | 3438±865 | 2460±1459 | 2985±1609 |
| **OH** | 4165±794 | 3214±1607 | 3195±797 | 2319±556 | 2005±220 |
|  | *Men* | | | | |
| **YE** | 5349±938 | 5171±2216 | 6499±1184 | 5480±1146 | 5706±2061 |
| **LLE** | 6713±1375* | 4657±2084 | 5598±1704 | 4003±1802 | 3906±1540 |
| LLE-P | 6645±1142 | 4614±2198 | 5359±1335 | 3878±966 | 3237±1242 |
| LLE-F | 6849±1857 | 4744±2078 | 6078±2329 | 4294±3126 | 5579±474 |
| **OH** | 5441±1278 | 3858±1451 | 5590±2372 | 5126±2826 | 4909±3352 |

Data presented as mean±SD. All units µm^2^. YE, young exercisers; LLE, lifelong exercisers;

OH, old healthy; MHC, myosin heavy chain. **P*<0.05 vs. other groups.
